# ADEPT - Abnormal Doppler Enteral Prescription Trial

**DOI:** 10.1186/1471-2431-9-63

**Published:** 2009-10-02

**Authors:** Alison Leaf, Jon Dorling, Steve Kempley, Kenny McCormick, Paul Mannix, Peter Brocklehurst

**Affiliations:** 1 Neonatal Unit, Southmead Hospital, Bristol, UK; 2 Neonatal Unit, Nottingham City Hospital, Hucknall Road, Nottingham, UK; 3 Neonatal Unit, Royal London Hospital, Whitechapel, London, UK; 4 Neonatal Unit, John Radcliffe Hospital, Headley Way, Headington, Oxford, UK; 5 Neonatal Unit, Northwick Park Hospital, Harrow, UK; 6 National Perinatal Epidemiology Unit, University of Oxford, Old Road Campus, Oxford, UK

## Abstract

**Background:**

Pregnancies complicated by abnormal umbilical artery Doppler blood flow patterns often result in the baby being born both preterm and growth-restricted. These babies are at high risk of milk intolerance and necrotising enterocolitis, as well as post-natal growth failure, and there is no clinical consensus about how best to feed them. Policies of both early milk feeding and late milk feeding are widely used. This randomised controlled trial aims to determine whether a policy of early initiation of milk feeds is beneficial compared with late initiation. Optimising neonatal feeding for this group of babies may have long-term health implications and if either of these policies is shown to be beneficial it can be immediately adopted into clinical practice.

**Methods and Design:**

Babies with gestational age below 35 weeks, and with birth weight below 10th centile for gestational age, will be randomly allocated to an "early" or "late" enteral feeding regimen, commencing milk feeds on day 2 and day 6 after birth, respectively. Feeds will be gradually increased over 9-13 days (depending on gestational age) using a schedule derived from those used in hospitals in the Eastern and South Western Regions of England, based on surveys of feeding practice. Primary outcome measures are time to establish full enteral feeding and necrotising enterocolitis; secondary outcomes include sepsis and growth. The target sample size is 400 babies. This sample size is large enough to detect a clinically meaningful difference of 3 days in time to establish full enteral feeds between the two feeding policies, with 90% power and a 5% 2-sided significance level. Initial recruitment period was 24 months, subsequently extended to 38 months.

**Discussion:**

There is limited evidence from randomised controlled trials on which to base decisions regarding feeding policy in high risk preterm infants. This multicentre trial will help to guide clinical practice and may also provide pointers for future research.

**Trial registration:**

Current Controlled Trials ISRCTN: 87351483

## Background

The purpose of this trial is to gain a better understanding of methods of establishing enteral feeding in high-risk preterm infants.

Preterm infants are at increased risk of adverse neonatal outcomes. At particular risk are those infants born after pregnancies in which Doppler studies of umbilical arterial wave forms reveal absent or reversed end diastolic flow velocities (AREDFV) [1]. This phenomenon occurs in approximately 6% of high risk pregnancies [2] and is believed to result from increased placental vascular resistance in response to both acute and chronic hypoxia. Lack of oxygen results in intrauterine growth restriction (IUGR) and the baby is often delivered preterm and small for gestational age. The prognosis is poor compared to those with normal antenatal Doppler studies [1-5]. In infants with abnormal umbilical artery Doppler blood flow velocities it has been shown that blood flow to the head tends to be preserved to support growth of the brain at the expense of blood flow to the abdomen and growth of visceral organs [3,6,7]. In the earlier stages of fetal hypoxia (before AREDFV occurs) the changes of cerebral redistribution may be seen, with widening of the ratio of blood flow velocity in the cerebral artery to that in the umbilical artery - the cerebro-placental ratio. An increase in this ratio has also been associated with increased perinatal morbidity [8-10].

Feeding babies born after AREDFV is a challenge: they are already under-nourished at birth, and good nutrition and growth is essential. However they frequently demonstrate intolerance of milk feeds and have been shown to have an increased incidence of necrotising enterocolitis (NEC) [1,3]. NEC is the commonest serious gastrointestinal emergency in neonatal intensive care units [11] and is associated with a high mortality and morbidity [12,13]. Extreme prematurity is the greatest risk factor, and whilst the specific aetiology is often not clear in individual babies, under perfusion and/or hypoxia of the gut are thought to be important predisposing factors [14]. Enteral feeding and bacterial invasion are commonly associated factors [14,15]. Reduced gut blood flow due to splanchnic vasoconstriction [1,3] may cause hypoxic-ischaemic damage to the intestine or its mucosa predisposing to NEC. Additionally, these conditions may affect normal motor, secretory and mucosal development so that the intestine is more susceptible to stasis, abnormal colonisation and bacterial invasion postnatally. IUGR is associated with bone marrow suppression and neutropenia in early postnatal life, which may also increase susceptibility to infective factors. Babies born after absent EDFV were found to have reduced flow velocities in the coeliac and superior mesenteric arteries compared with birthweight or gestational age matched controls [16]. Flow velocities improved but differences were still apparent on day 7 of life. A subsequent study showed impaired dynamic response in the superior mesenteric artery to the first milk feed in this group [17].

A recent meta-analysis [18] identified 14 observational studies [1,3,5,19-29] comparing the incidence of NEC in infants who had exhibited fetal AREDFV with a group of controls. Nine of these studies show an excess of NEC in the AREDFV infants, with an overall odds ratio for developing NEC of 2.13 (95% CI 1.49-3.03) compared with controls with forward fetal umbilical end-diastolic flow.

The incidence of NEC varies depending on the specific population. In the fourteen studies described above, including a total of 659 infants, the incidence varied between 0-59%, with an average of 12.9%. Among 2681 babies with birthweight 501-1500 grams born in, or transferred to, hospitals participating in the NICHD Neonatal Network between February 1988 and August 1989 the incidence of 'proven NEC' was 10.5%, with 'suspected NEC' at 17.2% [30]. The Vermont Oxford Network LBW Database (infants 401-1500 grams) shows an overall incidence of proven NEC (clinical and radiographic diagnosis) of 6% (VON Annual Reports 2002 and 2003). Analysis of the Network data previously showed an increased risk of NEC in babies with evidence of IUGR (birthweight below 10^th ^centile): OR 1.27 (95% CI 1.05-1.53) [31] but information on antenatal Doppler studies is not collected.

There is no consensus regarding how best to prevent NEC in small, preterm infants. Several strategies of poorly proven efficacy are in use, including delaying feeds, slowly increasing feeds, use of total parenteral nutrition (TPN) and prophylactic antibiotics [32-34]. A systematic review of prophylactic antibiotic use published in the Cochrane Library demonstrated a statistically significant reduction in NEC, but also a significant increase in the incidence of colonisation with resistant bacteria and concluded that there was insufficient evidence to support this approach in clinical practice [35].

The timing of introduction and rate of progression of milk feeds is an area of clinical uncertainty with arguments in favour of both early and late introduction of enteral feeds. Early introduction may improve nutrition and growth, but may increase the risk of NEC [30,32]. Conversely late introduction may be detrimental due to lack of stimulation of the gastrointestinal tract, resulting in villous atrophy and lack of hormone and enzyme production [30,36] and may not reduce the incidence of NEC [37,38]. Prolonged use of parenteral nutrition increases the risks of sepsis, cholestatic jaundice and vitamin and mineral deficiencies [39-41]. IUGR infants are at particularly high risk of parenteral nutrition-related liver disease [42].

The use of minimal enteral nutrition (MEN) (trophic feeds, gut-priming, non-nutritive feeding) has increasingly been used in the early feeding of preterm infants and appears to be well tolerated and beneficial in terms of gut motility, earlier establishment of substantive milk feeding and reduced cholestasis [43-49]. A systematic review published in the Cochrane Library [50] included eight randomised controlled trials and found that infants receiving MEN had an overall reduction in the time taken to achieve full enteral feeding and in the length of hospital stay. Regarding the effect on NEC the reviewers concluded that although no discernable effect was seen an increased risk of NEC could not be excluded - the risk ratio of NEC with MEN was 1.10 (95% CI 0.63-1.90).

Since this review was last updated in 1997, three further trials of MEN have been published. Van Elburg [51] specifically studied babies who were IUGR. Fifty six babies with birthweight below 2000 grams, and below the 10^th ^centile for gestational age, were randomised to receive either MEN (minimal enteral feeds - MEF) starting within 48 hours of birth, or no feeds for the first five days of life (NEF). Among the 42 who completed the trial there was no significant difference between groups in the primary outcome of intestinal permeability using a sugar absorbance test (p = 0.14). There was no difference in secondary outcomes of days to reach full feeds (p = 0.32), days to regain birthweight (p = 0.78) or NEC (0/20 cases in MEF group, 1/22 cases in NEF group (p = 0.76)). In those babies in whom it was measured (25/42), the ratio of umbilical artery to cerebral artery pulsatility index was not predictive of feeding tolerance (p = 0.55). In the two further trials McClure studied 100 infants, seeing 1 and 2 cases of NEC in MEN and control infants respectively [52]. Schanler's trial contained 171 infants, with 13 cases of NEC in the MEN group, compared to 10 cases in the control infants [53]. Combining these results with those of the Tyson meta-analysis, in 692 infants, NEC rates are similar at 10.5% for MEN and 9.4% for control infants (RR 1.07, 95% CI 0.84, 1.36).

The duration of MEN and subsequent rate of advancement is another area of uncertainty. Several studies have suggested that increasing feeds by 30-35 ml/kg/day is as safe as a slower rate of 15-20 ml/kg/day [54,55]. A trial of MEN versus progressive feeding in infants less than 32 weeks gestation was stopped early because of an increased incidence of NEC in the progressive feeding group [56]. In this study however feeds were started late in both groups (mean 10.3 and 9.3 days), and were given as two-hourly infusions followed by 2-hourly fasts. In addition breast milk fortifier was added when feeds of 120 ml/kg/day were reached and doubled when feeding volume reached 140 ml/kg/day.

There is thus no clear evidence to guide the choice of early or late introduction of enteral feeding in high risk IUGR infants. Practice varies widely as was discovered in surveys of neonatal units in two English health regions. In the Southwest enteral feeding was delayed in 9/12 hospitals for IUGR babies of less than 32 weeks gestation ('always' in three, 'usually' in six), and 'usually' in four hospitals for babies at 32-36 weeks. Feeds were delayed for less than five days in five hospitals, greater than five in one hospital and for variable duration in five. Abnormal Dopplers, polycythaemia, presence of umbilical artery catheter and the absence of breast milk made delay more likely. Within the 15 hospitals in the Eastern Region five units commenced feeds on day one, two delayed until day 7, with the remainder commencing feeds between day 2 and 5. The main reason cited for delaying feeds is to try to prevent NEC.

### Aim

We aim to evaluate the effects of an "early" enteral feeding regimen, starting milk feeds on day 2 after birth (between 24 and 48 hours of age) compared to one of "late" introduction of enteral feeds, starting feeds on day 6 after birth (between 120-143 hours of age) in a group of babies identified as being at high risk for NEC and milk intolerance by antenatal Doppler studies.

## Methods and Design

### Ethics Committee Approval

UK Multicentre Research Ethics Committee approval was initially granted in September 2005 with subsequent approvals for protocol amendment; this latest version was approved in January 2008.

### Eligibility and exclusions

#### Hospital eligibility

Hospitals will be eligible to participate in the ADEPT trial providing there are facilities for antenatal Doppler ultrasound of high risk pregnancies and that these are performed with appropriate filter settings, and facilities for neonatal high dependency care including parenteral nutrition.

#### Infant eligibility

Infants admitted to participating neonatal units and satisfying all of the following criteria may be recruited into the study:

1) Gestational age up to and including 34 weeks + 6 days (dated by antenatal ultrasound or clinically).

2) Antenatal ultrasound showing *either*

a) absent or reversed end diastolic flow velocities on at least 50% of the Doppler waveforms from the umbilical artery on at least one occasion during pregnancy

or

b) cerebral redistribution, defined as occurring when both the umbilical artery pulsatility index is greater than the 95^th ^centile and the middle cerebral artery pulsatility index is less that the 5^th ^centile for gestational age [9].

3) Small for gestational age (birth weight < 10^th ^centile for gestational age based on Child Growth Foundation Charts [57]).

4) Postnatal age 20-48 hours

Infants will be excluded if any of the following factors are present:

• major congenital abnormality including known chromosomal abnormality

• twin-twin transfusion

• intra-uterine transfusion or exchange transfusion

• Rhesus iso-immunisation

• significant multi-organ failure prior to trial entry

• inotropic drug support prior to trial entry

• already received any enteral feeding

#### Recruitment and randomisation

Informed written consent for the trial will be obtained from the parents in the first 2 days after birth. This will preferably be in the first 24 hours after birth. The parents will be given a verbal explanation of the study and a written information sheet, and will have an opportunity to discuss participation with the recruiting clinician. If consent is given, and providing no contra-indications occur, the baby should then be randomised between 20 and 48 hours of age. Babies will be randomised to study groups by a central randomisation service, based at the National Perinatal Epidemiology Unit (NPEU), University of Oxford. The randomisation service (website with telephone back-up facility) will request details of the baby and, if eligible, will provide a random allocation to one of the study groups, either "early" or "late" commencement of enteral feeds. The program will use minimisation to ensure balance between the groups with respect to hospital of recruitment, gestational age (< 29 completed weeks or ≥ 29 completed weeks) and type of Doppler abnormality (AREDFV or cerebral redistribution).

### Interventions

Babies will be randomly allocated to an "early" or "late" enteral feeding regimen. These will start milk feeds on day 2 and day 6 after birth, respectively. The regimens for the two groups are based on those currently used in hospitals in the Eastern and South Western Regions, according to a survey of practice carried out in 1999.

The two regimens are as follows:

### 'Early' and 'Late' Feeding Regimens: see Table 1

**Table 1:** 'Early' and 'Late' Feeding Regimens

**Feed regimen**	**'early'**	**'late'**
0-24 hours: day 1	Nil by mouth	Nil by mouth
24-48 hours: day 2	Start milk feeds according to tables 2 & 3	Nil by mouth
48-119 hours: day 3-5	Progress with feeding according to tables 2 & 3	Nil by mouth
120-143 hours: day 6	Progress with feeding according to tables 2 & 3	Start milk feeds according to tables 2 & 3
144 hours onwards - day 7+	Progress with feeding according to tables 2 & 3	Progress with feeding according to tables 2 & 3

For the purposes of the study the choice of milk recommended to mothers to feed their baby should be, in descending order of preference: mother's own breast milk, donated breast milk, infant formula (all dependent on availability). Whether preterm or term formula is the formula given initially will be at the discretion of the local clinician but the recommendation would be for infants with gestation less than 34 weeks to be fed preterm formula within one week of milk commencement. The final choice of which milk is used will rest with the infant's mother. Breast milk fortification may be considered if additional nutritional support is required once the baby is tolerating full milk feeds of breast milk of ≥ 150 ml/kg/day.

The feeding schedule of each group should be followed regardless of the type of milk available, ventilation status, or presence of an UAC unless specifically requested by the local clinician. The decision to withhold feeds or deviate from the feeding schedule in Tables 2 and 3, because of apparent feed intolerance or clinical deterioration, will also remain at the local clinician's discretion. Gastric residuals are not uncommon in preterm infants [58]. Providing the infant is well and has no abnormal abdominal signs it is usually safe to continue with enteral feeds when gastric aspirate is 2-3 ml or less (2 ml in a baby of less than 750 grams birth weight). If feed volumes are withheld or there is any deviation from the schedule in Tables 2 and 3 then the clinician is free to either start again from day 1, re-start at the volume previously tolerated then increase as scheduled daily, or hold for one or more days at a certain volume and then increase as scheduled.

**Table 2:** Feeding schedule - ml/kg/HOUR

**Day of feeding**	**Volume of milk according to birth weight (ml/kg/HOUR)**				
	
	**< 600 g**	**600-749 g**	**750-999 g**	**1000-1249 g**	**≥ 1250 g**
1	0.5	0.5	0.5	0.5	1.0

2	0.5	0.5	0.5	1.0	1.5

3	0.5	1.0	1.0	1.5	2.0

4	1.0	1.5	1.5	2.0	2.5

5	1.5	2.0	2.0	2.5	3.0

6	2.0	2.5	2.5	3.0	3.5

7	2.5	3.0	3.0	3.5	**4.0 - 4.5**

8	3.0	3.5	3.5	**4.0 - 4.5**	**5.0 - 5.5**

9	3.5	4.0	**4.0 - 4.5**	**5.0 - 5.5**	**6.0 - 6.25**

10	4.0	**4.5 - 5.0**	**5.0 - 5.5**	**6.0 - 6.25**	

11	**4.5 - 5.0**	**5.5 - 6.0**	**6.0 - 6.25**		

12	**5.5 - 6.0**	**6.25**			

13	**6.25**				

14	Increase as required				

(NB feed advancement schedule is *the same *for babies in EARLY or LATE groups: only the timing of initiation of feeds differs)

Where 2 numbers are in a cell separated by a hyphen the first number indicates hourly volume/kg to feed for the first 12 hours of each 24 hour period. The second number in each cell indicates hourly volume/kg to feed for the second 12 hours of each 24 hour period.

Feeds should be given hourly or continuously. However, if there are longer intervals between feeds, e.g. 2 hourly feeds, then the milk volume should be increased accordingly.

Light grey background = feeds increase by 0.5 ml/kg every 12 hours.

Dark grey background = feed volume reached 150 ml/kg/day (6.25 ml/kg/hour).

Feeds can be increased as required.

**Table 3:** Feeding schedule - ml/kg/DAY (NB feed advancement schedule is *the same *for babies in EARLY or LATE groups: only the timing of initiation of feeds differs).

**Day of feeding**	**Volume of milk according to birth weight (ml/kg/DAY)**				
	
	**<600 g**	**600-749 g**	**750-999 g**	**1000-1249 g**	**≥ 1250 g**
1	12	12	12	12	24

2	12	12	12	24	36

3	12	24	24	36	48

4	24	36	36	48	60

5	36	48	48	60	72

6	48	60	60	72	84

7	60	72	72	84	**96 - 108**

8	72	84	84	**96 - 108**	**120-132**

9	84	96	**96-108**	**120-132**	**144-150**

10	96	**108-120**	**120-132**	**144-150**	

11	**108-120**	**132-144**	**144-150**		

12	**132-144**	**150**			

13	**150**				

14	Increase as required				

### Other clinical management

Clinical management will include commencement of intravenous parenteral nutrition (glucose, amino acids and intralipid) by the second or third day after birth for all babies. All other aspects of care will be according to local routine practice.

### Outcomes

#### Primary outcome measures

1. Age in days at which full enteral feeding sustained for 72 hours was reached.

2. Necrotising enterocolitis, stage I, II or III [11] (Appendix 1)

#### Secondary outcome measures

• Death before hospital discharge

• Duration of hospital stay

• Duration of intensive and high dependency care (Appendix 2)

• Duration of parenteral nutrition

• Change in Z score for weight and head circumference from birth to 36 weeks post-

conceptional age and from birth to discharge

• In continuous supplemental oxygen at 36 weeks post-menstrual age

• Confirmed bacterial sepsis (Appendix 3)

• Gastrointestinal perforation

• Gastrointestinal surgery

• Cholestasis (defined as >25 μmol/l conjugated fraction of serum bilirubin)

• Patent ductus arteriosus requiring pharmacological or surgical treatment

• Cranial ultrasound abnormality

• Type of milk at discharge

• On oxygen therapy at discharge

No additional blood tests will be performed because of this study.

### Data collection

Data will be collected at trial entry, during the infant's stay in the neonatal unit, and at discharge. At trial entry baseline data and eligibility information will be collected and returned to the co-ordinating centre. Information collected during the infant's stay in the neonatal unit will include enteral feeding history (to assess compliance) and other treatments given (to assess whether interventions are used differentially in the two groups). This is important because caregivers will not be blinded to the randomised allocations. Outcome data will be recorded on a form competed by clinicians at discharge. To facilitate later tracing for follow-up and enable notification of any deaths, all infants recruited to the study will be flagged at the NHS Central Register.

All data required for trial analysis will be routinely collected in medical and nursing charts.

Perinatal and early neonatal data will be submitted to the ADEPT Co-ordinating Centre at time of trial entry. Subsequent neonatal data and outcome data will be submitted at discharge *and on transfer to a different neonatal unit if applicable*. Growth data - weight and head circumference - will be collected at 36 weeks post-menstrual age *and *at discharge.

### Serious Adverse Event and Suspected Unexpected Serious Adverse Reaction reporting

Serious Adverse Events and Suspected Unexpected Serious Adverse Reactions should be reported to the ADEPT co-ordinating office within 48 hours. The co-ordinating office will then report it to the Chair of the DMEC and the MREC with a summary of the previously reported events within 15 days. As both early and late feeding policies are already used in different hospitals within the UK there are no SAEs which would be anticipated as a unique consequence of participation in the trial. We would however expect the following to be reported:

• All deaths

• Severe central venous line complication: cardiac tamponade, major vessel thrombosis

Any other serious unexpected adverse events

### Statistical analysis

Analysis will be by intention to treat i.e. all babies will be analysed in their allocated groups, regardless of the treatment they actually received. For dichotomous outcomes, relative risks and 95% confidence intervals will be calculated. For continuous outcomes, differences in means or differences in medians (depending on the distribution of the data) will be calculated along with 95% confidence intervals. Analysis of time to event outcomes such as time to reach full enteral feeding and duration of stay in hospital will use survival analysis techniques.

Two pre-specified subgroup analyses will be conducted, stratifying by (a) gestational age at birth (< or ≥ 29 weeks) and (b) type of Doppler abnormality (AREDFV or cerebral redistribution). Statistical tests of interaction will be used for the subgroup analyses. Interim analyses will be conducted at least once per year during the period of recruitment, and reviewed in confidence by a Data Monitoring Committee.

### Interim analysis

A Data Monitoring Committee (DMC), independent of the trial investigators, has been established. The DMC terms of reference, including the statistical stopping rules, have been agreed and are documented in the ADEPT DMC Charter. During the period of recruitment to the trial, interim analyses will be supplied in strict confidence to the DMC as frequently as they request. Meetings of the committee will be at least once a year as considered appropriate by the Chair.

In the light of interim data, and other evidence from relevant studies (including updated overviews of the relevant randomised controlled trials), the DMC will inform the Trial Steering Committee, if in their view there is proof beyond reasonable doubt that the data indicate that any part of the protocol under investigation is either clearly indicated or contra-indicated, either for all or for a particular subgroup of trial participants.

Unless modification or cessation of the protocol is recommended by the DMC, the Trial Steering Committee, collaborators and administrative staff (except those who supply the confidential information) will remain ignorant of the results of the interim analysis. Collaborators and all others associated with the study may write through the study co-ordinating centre to the DMC, to draw attention to any concern they may have about the possibility of harm arising from the treatment under study, or about any other matters that may be relevant.

### Membership of the Data Monitoring Committee

Professor Richard Cooke (Chair)

Dr Simon Newell

Dr John Puntis

Ms Ly-Mee Yu

### Establishment of a blinded endpoint committee

As a result of central monitoring the trial data, it has become clear that the primary end point has not been achieved in a small number of cases. There are two main reasons for this:

i) the introduction of breast-feeding

ii) completion of the feeding log has stopped prematurely in the mistaken belief that the primary endpoint has been reached

Where there is uncertainty as to whether the primary endpoint has been reached, a committee has been established to consider each case and recommend an appropriate course of action. The committee will be blind to allocation.

### Membership of the Blinded Endpoint Committee

Dr Alison Leaf.

Dr Kenny McCormick.

Dr Steve Kempley.

### Sample size

Using unpublished data from the Eastern Region Very Low Birthweight database, which suggests a standard deviation of 9 days in the time taken to reach full enteral feeding, about 380 infants will be required to show a difference of 3 days in this outcome with 90% power. The incidence of NEC is about 15% in this population and a sample of 400 would be sufficient to show a reduction to 7.5% with 60% power. NEC is retained as a primary outcome because of its clinical importance for this group of babies. We acknowledge that the power to detect relatively small differences will be limited. The target sample size is 400 babies, to be recruited over 24 months.

### Feasibility

There is little information on the number of babies that may be eligible for this trial. We estimate that each participating hospital may have around 10-30 eligible babies per year depending on obstetric case mix. If an average hospital has 15 eligible babies per year and a third of these can be recruited (i.e. 5 recruits/hospital/year), around 40 hospitals will need to participate to complete recruitment of 400 babies in 2 years.

### Organisation

The trial will be overseen by a Trial Steering Committee, consisting of the Investigators and the project team at NPEU plus independent members. This group will meet regularly throughout the trial to review progress and resolve problems, receive reports from the Data Monitoring Committee and take decisions about the trial's conduct.

The trial co-ordinating centre will be at the NPEU, where the Trial Co-ordinator will be based. The NPEU will be responsible for day to day co-ordination of the trial, including recruitment of hospitals to the study, programming, data management, and statistical analysis.

### Membership of the Trial Steering Committee

The Membership of the Trial Steering Committee is listed in Table 4

**Table 4:** Membership of the Trial Steering Committee

Professor Zarko Alfirevic	Consultant in Obstetrics & Gynaecology	Chair
Dr Andrew Ewer	Consultant Neonatologist	Independent member
Ms Pauline Fellows	NSC Neonatal Project Facilitator	Independent member
Professor Khalid Khan	Consultant in Obstetrics & Gynaecology	Independent member
Dr Alison Leaf	Consultant Neonatologist & Speciality Director	Clinical Lead
Professor Peter Brocklehurst	Director, NPEU, University of Oxford	Chief Investigator
Dr Steve Kempley	Consultant Neonatologist	Clinical Investigator
Dr Paul Mannix	Consultant Neonatologist	Clinical Investigator
Dr Jon Dorling	Consultant Neonatologist	Clinical Investigator
Dr Kenny McCormick	Consultant Neonatologist	Clinical Investigator

### Local co-ordination

Each participating centre will identify a site specific principal investigator to act as local co-ordinator for that centre. Their responsibilities will be to:

i) be familiar with the trial

ii) liaise with the Trial Co-ordinating centre in Oxford

iii) ensure that all staff involved in the care of eligible babies are informed about the trial

iv) ensure that mechanisms for recruitment of eligible babies (including information material) are in place, monitor their effectiveness, and discuss reasons for the non-recruitment with relevant staff

v) ensure that supplies of data collection forms are available, that they are completed and returned to the Trial Co-ordinating centre promptly, and to deal with any queries arising

vi) notify the Trial Co-ordinating centre of any serious adverse events

vii) make all data available for verification, audit and inspection purposes as necessary

viii) ensure that the confidentiality of all information about trial participants is respected by all persons.

## Competing interests

The authors declare that they have no competing interests.

## Authors' contributions

All authors are members of the ADEPT Study Investigators' Group, representing the ADEPT Study Collaborators, and were involved in the conception and design of the study. All authors edited the manuscript, read and approved the final protocol.

## Appendix 1: Modified Bell's criteria from Walsh MC et al 1986

### Stage IA - Suspected NEC

Systemic signs: temperature instability, apnoea, bradycardia, lethargy

Intestinal signs: Elevated pre-gavage residuals, mild abdominal distension, emesis, haem-positive stools

Radiologic signs: Normal or intestinal dilatation, mild ileus

### Stage IB - Suspected NEC

Systemic signs: Same as stage IA

Intestinal signs: bright red blood from rectum

Radiologic signs: same as stage IA

### Stage IIA - Definite NEC (mildly ill)

Systemic signs: Same as stage IA

Intestinal signs: Same as stage IA, plus absent bowel sounds, +/- abdominal tenderness

Radiologic signs: intestinal dilatation, ileus, pneumatosis intestinalis

### Stage IIB - Definite NEC (moderately ill)

Systemic signs: Same as stage IIA, plus mild metabolic acidosis, mild thrombocytopenia

Intestinal signs: Same as stage IIA, plus absent bowel sounds, definite abdominal tenderness, +/- abdominal cellulitis or right lower quadrant mass

Radiologic signs: Same as stage IIA plus portal vein gas, +/- ascites

### Stage IIIA - Advanced NEC (severely ill, bowel intact)

Systemic signs: Same as stage IIB, plus hypotension, bradycardia, severe apnea, combined respiratory and metabolic acidosis, DIC, neutropenia

Intestinal signs: Same as stage IIB, plus signs of generalized peritonitis, marked tenderness, and distension of abdomen

Radiographic signs: Same as stage IIB, plus definite ascites

### Stage IIIB - Advanced NEC (severely ill, bowel perforated)

Systemic signs: Same as stage IIIA

Intestinal signs: Same as IIIA

Radiologic signs: Same as stage IIB, plus pneumoperitoneum

## Appendix 2: Definition of levels of neonatal intensive care (BAPM 2001)

### Intensive Care includes babies

Receiving any respiratory support via a tracheal tube and in the first 24 hours after its withdrawal

Receiving NCPAP for any part of the day and less than five days old

Below 1000 g current weight and receiving NCPAP for any part of the day and for 24 hours after withdrawal

Less than 29 weeks gestational age and less than 48 hours old

Requiring major emergency surgery, for the pre-operative period and post-operatively for 24 hours

On the day of death

Requiring complex clinical procedures:

• Full exchange transfusion

• Peritioneal dialysis

• Infusion of an inotrope, pulmonary vasodilator or prostaglandin and for 24 hours afterwards

Any other very unstable baby considered by the nurse-in-charge to need 1:1 nursing

### High dependency care includes babies

Receiving NCPAP for any part of the day and not fulfilling any of the criteria for intensive care

Below 1000 g current weight and not fulfilling any of the criteria for intensive care

Requiring parenteral nutrition

Having convulsions

Receiving oxygen therapy and below 1500 g current weight

Requiring treatment for neonatal abstinence syndrome

Requiring specified procedures that do not fulfil any criteria for intensive care:

• Care of an intra-arterial catheter or chest drain

• Partial exchange transfusion

• Tracheostomy care until supervised by the parent

Requiring frequent stimulation for severe apnoea

## Appendix 3 - Definition of infection

Symptomatic baby with positive culture of blood, CSF, or other normally sterile site, and with haematological markers of infection including one or more of the following: raised CRP, high or low white blood cell count, thrombocytopenia.

## Pre-publication history

The pre-publication history for this paper can be accessed here:


